# Mass spectrometry-based identification of a B-cell maturation antigen-derived T-cell epitope for antigen-specific immunotherapy of multiple myeloma

**DOI:** 10.1038/s41408-020-0288-3

**Published:** 2020-02-28

**Authors:** Tatjana Bilich, Annika Nelde, Jens Bauer, Simon Walz, Malte Roerden, Helmut R. Salih, Katja Weisel, Britta Besemer, Ana Marcu, Maren Lübke, Juliane Schuhmacher, Marian C. Neidert, Hans-Georg Rammensee, Stefan Stevanović, Juliane S. Walz

**Affiliations:** 1 University Hospital Tübingen, Clinical Collaboration Unit Translational Immunology, German Cancer Consortium (DKTK), Tübingen, Germany; 2 University of Tübingen, Institute for Cell Biology, Department of Immunology, Tübingen, Germany; 3 University Hospital Tübingen, Department of Urology, Tübingen, Germany; 4 University Hospital Tübingen, Department of Hematology and Oncology, Tübingen, Germany; 5 University Hospital Hamburg-Eppendorf, Department of Oncology, Hamburg-Eppendorf, Germany; 6 University Hospital Zurich and University of Zurich, Department of Neurosurgery, Clinical Neuroscience Center, Zurich, Switzerland; 7 German Cancer Consortium (DKTK), DKFZ partner site Tübingen, Tübingen, Germany

**Keywords:** Myeloma, Tumour immunology, Myeloma, Tumour immunology, Immunotherapy

## Abstract

The B-cell maturation antigen (BCMA) is currently being evaluated as promising tumor-associated surface antigen for T-cell-based immunotherapy approaches, such as CAR T cells and bispecific antibodies, in multiple myeloma (MM). Cytotoxic T cells bearing BCMA-specific T-cell receptors might further allow targeting HLA-presented antigens derived from the intracellular domain of BCMA. By analyzing a mass spectrometry-acquired immunopeptidome dataset of primary MM samples and MM cell lines for BCMA-derived HLA ligands, we identified the naturally presented HLA-B*18-restricted ligand P(BCMA)_B*18_. Additionally, P(BCMA)_B*18_ was identified on primary CLL samples, thereby expanding the range for possible applications. P(BCMA)_B*18_ induced multifunctional BCMA-specific cells de novo from naïve CD8^+^ T cells of healthy volunteers. These T cells exhibited antigen-specific lysis of autologous peptide-loaded cells. Even in the immunosuppressive context of MM, we detected spontaneous memory T-cell responses against P(BCMA)_B*18_ in patients. By applying CTLA-4 and PD-1 inhibition in vitro we induced multifunctional P(BCMA)_B*18_-specific CD8^+^ T cells in MM patients lacking preexisting BCMA-directed immune responses. Finally, we could show antigen-specific lysis of autologous peptide-loaded target cells and even MM.1S cells naturally presenting P(BCMA)_B*18_ using patient-derived P(BCMA)_B*18_-specific T cells. Hence, this BCMA-derived T-cell epitope represents a promising target for T-cell-based immunotherapy and monitoring following immunotherapy in B-cell malignancy patients.

## Introduction

T-cell-based immunotherapies for multiple myeloma (MM) are on the advance, further improving the outcome of this still incurable disease. Beside strategies like allogeneic stem cell transplantation^1^, donor lymphocyte infusion^2^, immunomodulatory drugs^3^, and immune checkpoint inhibitors^4^, that induce a fairly general T-cell activation, several advanced approaches for targeting myeloma cells more specifically are in development. Such antigen-specific immunotherapy approaches comprise mono- and bispecific antibodies^5,6^, chimeric antigen receptor (CAR) T cells^7^, T-cell receptor (TCR)-engineered T cells^8^, and dendritic cell (DC)- or peptide-based vaccines^9,10^. A main prerequisite for such approaches is the selection of feasible targets for MM-directed T-cell responses. Ideally, such targets should be presented both, exclusively and frequently on myeloma cells. A particularly promising target for antigen-specific immunotherapy in MM and other B-cell malignancies is the B-cell maturation antigen (BCMA, TNFRSF17, CD269), a member of the tumor necrosis receptor superfamily. BCMA is a preferentially B-lineage-restricted differentiation transmembrane protein with exclusive presentation on myeloma cells, plasma blasts, differentiated plasma cells, and late memory B cells^6^.

Currently, several therapeutic approaches targeting BCMA are being evaluated in preclinical settings and clinical trials including CAR T cells^7^, bispecific antibodies and other T-cell engagers^6,11^, antibody-drug conjugates^12^, or immunotoxins^13^. Particularly BCMA-targeting CAR T cells showed promising results in heavily pretreated and refractory MM patients with an overall response rate of 81%^7^. However, despite these encouraging results, potential toxicities and immense costs should be taken into account, in particular prior to application of such immunotherapy approaches in elderly and comorbid MM patients or larger patient cohorts at an earlier disease setting^14,15^. Furthermore, all these substances are targeting the extracellular domain of BCMA with an associated risk of surface antigen loss^16^. Cytotoxic T cells bearing BCMA-specific TCRs might be an alternative approach to target MM cells. Such TCRs can also recognize intracellular proteins or domains, which are processed and presented *via* human leukocyte antigen (HLA) molecules on the surface of tumor cells^17^. Antigen-specific T cells can either be induced in vivo by low side effect vaccination-based approaches or generated ex vivo as TCR-engineered cells. The main prerequisite for these approaches is the identification and characterization of naturally presented HLA-restricted peptides, which can serve as target structures for T cells^18^. In a previous study, we characterized the naturally presented immunopeptidome of MM using a mass spectrometry (MS)-based approach and identified several novel MM-associated antigens^19^. Here, we evaluated this dataset for the presence of BCMA-derived peptides to provide a proof of concept for the feasibility to identify and target naturally presented T-cell epitopes from intracellular domains of highly promising tumor surface antigens.

## Results

### MS-based identification of BCMA-derived HLA-presented peptides in MM

**Fig. 1:**
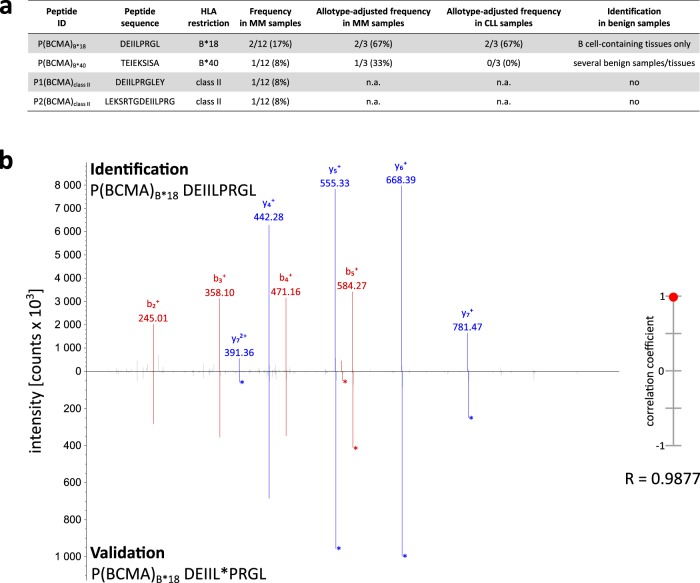
Identification of BCMA-derived peptides and validation of P(BCMA)_B*18_ using a synthetic isotope-labeled peptide. **a** Identified BCMA-derived HLA-presented peptides with their respective sequence, HLA restriction, their total and allotype-adjusted frequency in the immunopeptidomes of the MM and CLL cohort, as well as their occurrence in the HLA peptidome of benign tissues. **b** Validation of the experimentally eluted P(BCMA)_B*18_ peptide using the corresponding synthetic isotope-labeled peptide. Comparison of the fragment spectrum (*m/z* on the *x*-axis) of the P(BCMA)_B*18_ peptide eluted from a primary MM patient sample (identification) with its corresponding synthetic peptide (validation). The spectrum of the synthetic peptide is mirrored on the *x*-axis. Identified b- and y-ions are marked in red and blue, respectively. Ions containing the isotope-labeled amino acid are marked with asterisks. The calculated spectral correlation coefficient is depicted on the right graph. ID identification, MM multiple myeloma, CLL chronic lymphocytic leukemia, n.a. not available.

Previously acquired MS datasets^19,20^ of primary MM samples and MM cell lines (MCLs) were reprocessed using the search engine SequestHT and evaluated for the presence of naturally presented BCMA-derived peptides. Analysis of the immunopeptidome of seven primary MM samples and five MCLs revealed a total of 17 633 unique HLA class I ligands from 7 627 different source proteins as well as 9 482 unique HLA class II peptides from 2 371 source proteins. We identified two BCMA-derived HLA class I-restricted ligands, both derived from its intracellular domain (Fig. 1a). The HLA-B*18-restricted peptide DEIILPRGL, referred to as P(BCMA)_B*18_, was identified in 17% (2/12 samples, one primary MM patient sample and the MCL MM.1S) of the analyzed MM immunopeptidomes with a remarkably high allotype-adjusted frequency of 67% (2/3 HLA-B*18^+^ samples). Notably, P(BCMA)_B*18_ showed MM- and B-lineage-associated presentation and was solely detected on 1/5 benign B-cell (20%) and 2/17 benign lymph node samples (12%) according to our extensive benign immunopeptidome database (149 297 HLA class I ligands; 17 093 source proteins; 404 samples from various tissues). Additionally, P(BCMA)_B*18_ could also be identified in the immunopeptidome of 2/3 (67%) primary HLA-B*18^+^ chronic lymphocytic leukemia (CLL) samples^21^. In contrast, the HLA-B*40-restricted P(BCMA)_B*40_ ligand TEIEKSISA was detected solely in 1/12 (8%) MM-derived samples with an allotype-adjusted frequency of 33% (1/3 HLA-B*40^+^ samples) but displayed no selective MM-association due to its representation in a variety of benign tissues. Furthermore, we identified two HLA class II-restricted BCMA-derived antigens that showed MM-exclusive presentation according to our benign HLA class II immunopeptidome database (214 908 HLA class II peptides; 15 840 source proteins; 366 samples from various tissues). However, these HLA class II-restricted BCMA-derived peptides were both detected only in MCLs but not in primary MM samples with a low representation frequency of 8% (1/12 samples) in our MM cohort.

Therefore, we selected the P(BCMA)_B*18_ peptide due to its MM-association and the high representation frequency for further immunological characterization. Prior to immunogenicity testing, we validated the experimentally acquired spectrum of P(BCMA)_B*18_ by comparison of MS/MS spectra as well as of the reversed-phase retention times of the precursor ions using an isotope-labeled synthetic peptide (Fig. 1b).

### P(BCMA)_B*18_ induced multifunctional peptide-specific T cells in healthy volunteers in vitro

**Fig. 2:**
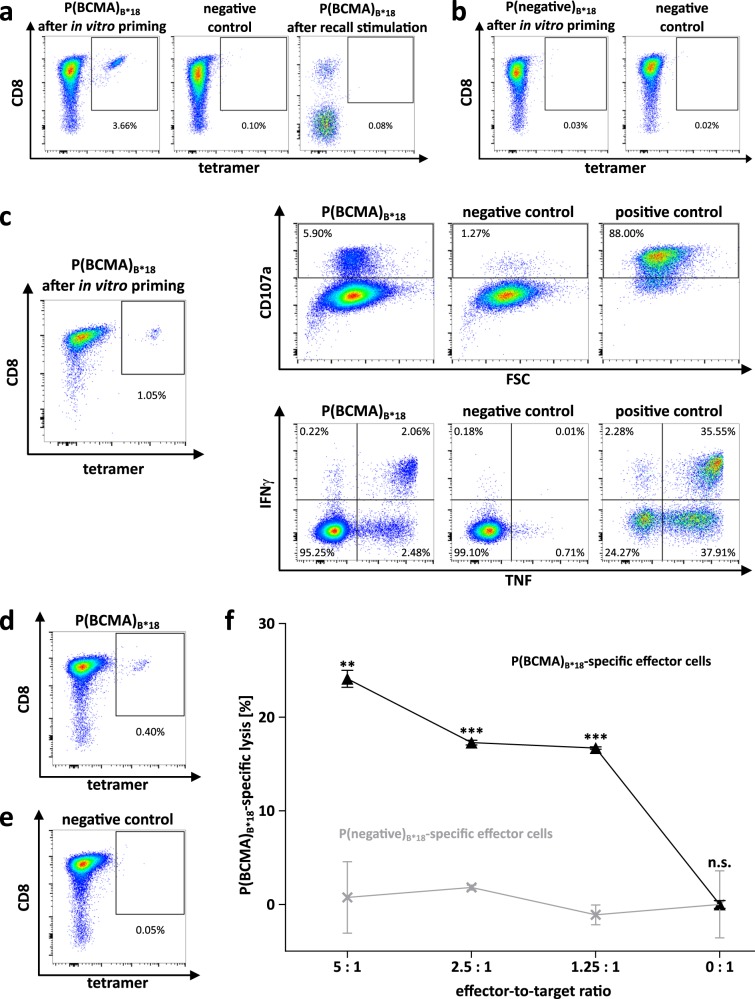
Induction and functional characterization of P(BCMA)_B*18_-specific CD8^+^ T cells from HVs. **a** Naïve CD8^+^ T cells from HVs were primed in vitro using aAPCs. Graphs show single, viable cells stained for CD8 and PE-conjugated multimers of indicated specificity. Tetramer staining was performed after four stimulation cycles with peptide-loaded aAPCs. The left panel shows P(BCMA)_B*18_-tetramer staining. The middle panel (negative control) depicts P(BCMA)_B*18_-tetramer staining of T cells from the same donor primed with a control peptide. The right panel shows PBMCs from the same donor that were tested negative for preexisting memory T cells following 12-day recall stimulation. **b** Tetramer staining after four stimulation cycles with negative control peptide-loaded aAPCs. **c** Functional characterization of P(BCMA)_B*18_-specific CD8^+^ T cells by intracellular cytokine staining. Representative example of IFNγ and TNF production as well as CD107a expression after stimulation of P(BCMA)_B*18_-specific CD8^+^ T cells with the P(BCMA)_B*18_ peptide compared to a negative control peptide. PMA and ionomycin served as positive control. **d–f** Cytotoxicity of P(BCMA)_B*18_-specific effector T cells analyzed in a VITAL assay with in vitro primed CD8^+^ T cells. **d**, **e** Tetramer staining of polyclonal effector cells prior to the VITAL assay determined the amount of P(BCMA)_B*18_-specific effector cells in the **d** population of successfully P(BCMA)_B*18_-primed CD8^+^ T cells and in the **e** population of control cells from the same donor primed with an HLA-matched control peptide. **f** Cell lysis by P(BCMA)_B*18_-specific effector T cells (black) of P(BCMA)_B*18_-loaded autologous CD8^-^ target cells at various effector-to-target cell ratios in comparison to negative control peptide-loaded CD8^-^ target cells. P(BCMA)_B*18_-unspecific effector cells (grey) showed no peptide-specific lysis of the same targets. Results are shown as mean ± SEM for three independent technical replicates. Significance was determined using two-tailed paired student’s *t*-test. FSC forward scatter, n.s. not significant, **p < 0.01, ***p < 0.001.

To assess the immunogenicity of P(BCMA)_B*18_, we performed in vitro artificial antigen-presenting cell (aAPC)-based priming experiments using CD8^+^ T cells of healthy volunteers (HVs). Effective de novo priming and expansion of antigen-specific T cells was observed in 100% of analyzed HVs (*n* = 10) with frequencies of peptide-specific T cells ranging from 0.1–7.9% (mean 0.9%) within the viable CD8^+^ T-cell population (Fig. 2a, Supplemental Table 1). The de novo induction of peptide-specific cells was further proven by the lack of preexisting memory T-cell responses after 12-day recall stimulation (Fig. 2a). Priming experiments with a control peptide frequently presented on HLA-B*18 in both, tumor and benign tissues (peptide presentation > 90% in HLA-matched sources), confirmed MM-specificity of the induced T-cell responses (Fig. 2b, Supplemental Table 2). Furthermore, multifunctionality of the induced P(BCMA)_B*18_-specific T cells could be demonstrated in 6/6 HV samples. Using intracellular cytokine staining (ICS), we observed a significant production of IFNγ and TNF as well as upregulation of the degranulation marker CD107a upon P(BCMA)_B*18_ stimulation (Fig. 2c). Moreover, cytotoxicity assays with polyclonal P(BCMA)_B*18_-specific effector T cells revealed their capacity to induce antigen-specific lysis of autologous CD8^−^ peptide-loaded target cells (Fig. 2d–f).

### Detection of preexisting P(BCMA)_B*18_-specific memory T-cell responses in MM patients

**Fig. 3:**
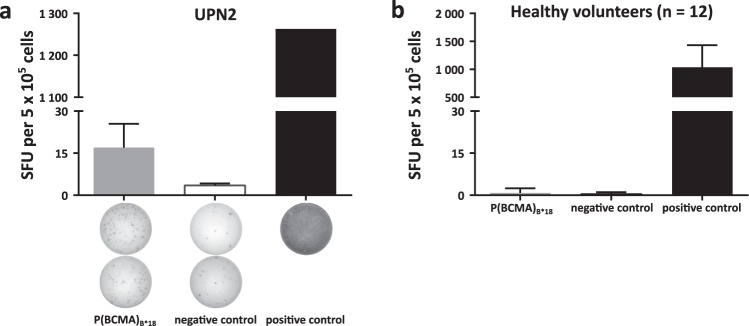
Preexisting memory T-cell responses directed against BCMA detected in IFNγ ELISPOT assays. **a**, **b** Memory T-cell responses directed against P(BCMA)_B*18_ were evaluated in IFNγ ELISPOT assays after 12-day recall stimulation using PBMCs of **a** MM patients or **b** HVs. PHA was used as positive control. The peptide DEVRTLTY served as negative control. Data are expressed as mean ± SD of two independent technical replicates for the MM patient and as mean ± SD of all 12 HVs analyzed in two independent technical replicates each. UPN uniform patient number, HVs healthy volunteers, SFU spot forming unit.

We further evaluated the existence of spontaneous preexisting memory T-cell responses directed against P(BCMA)_B*18_ in IFNγ enzyme-linked immunospot (ELISPOT) assays using peripheral blood mononuclear cells (PBMCs) of HLA-B*18^+^ MM patients (Supplemental Table 3). We observed P(BCMA)_B*18_-induced IFNγ secretion in 1/4 (25%) samples of MM patients (Fig. 3a). Notably, we could not detect any preexisting immune responses against P(BCMA)_B*18_ in samples from HVs (*n* = 12, Fig. 3b), further confirming the MM-specificity of P(BCMA)_B*18_-directed immune responses.

### Combination of immune checkpoint inhibitors and P(BCMA)_B*18_ using cells of MM patients induced peptide-specific T cells with anti-myeloma activity

**Fig. 4:**
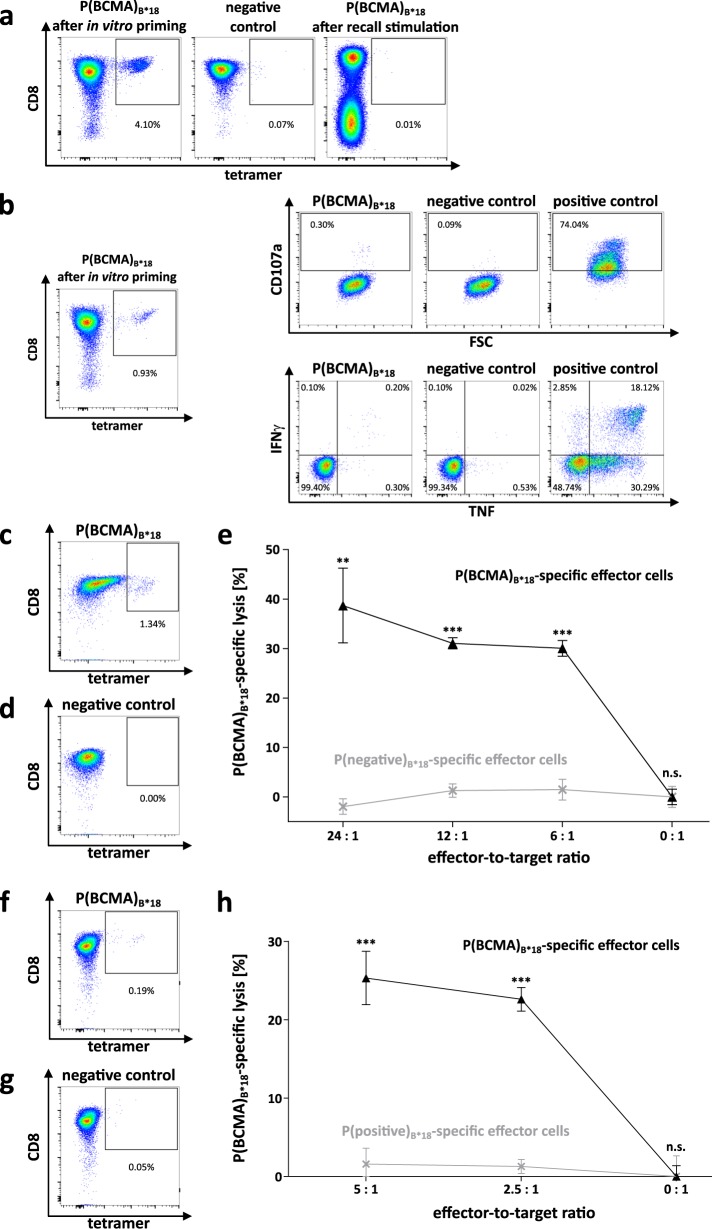
Induction and functional characterization of P(BCMA)_B*18_-specific CD8^+^ T cells from a myeloma patient. **a** Naïve CD8^+^ T cells from UPN1 were stimulated four times with peptide-loaded aAPCs in addition to PD-1 and CTLA-4 blocking antibodies. Graphs show single, viable cells stained for CD8 and PE-conjugated multimers of indicated specificity. The left panel shows P(BCMA)_B*18_-tetramer staining. The middle panel (negative control) depicts P(BCMA)_B*18_-tetramer staining of T cells from the same patient primed with a control peptide. The right panel shows PBMCs from the same patient that were tested negative for preexisting memory T cells following 12-day recall stimulation. **b** Example of IFNγ and TNF production as well as CD107a expression following stimulation with the P(BCMA)_B*18_ peptide compared to the negative control peptide using peptide-specific cells of UPN1. PMA and ionomycin served as positive control. **c**, **d**, **f**, **g** Tetramer staining of polyclonal effector cells prior to the VITAL assay determined the amount of P(BCMA)_B*18_-specific cells in the **c**, **f** population of successfully P(BCMA)_B*18_-primed CD8^+^ T cells and in the **d**, **g** population of cells from the same MM patient primed with an HLA-matched control peptide. **e** Cell lysis by P(BCMA)_B*18_-specific effector T cells (black) of P(BCMA)_B*18_-loaded autologous CD8^-^ target cells at various effector-to-target cell ratios in comparison to P(negative)_B*18_ control peptide-loaded CD8^-^ target cells. P(BCMA)_B*18_-unspecific effector cells (gray) showed no peptide-specific lysis of the same targets. Results are shown as mean ± SEM for three independent technical replicates. **h** Cell lysis by P(BCMA)_B*18_-specific effector cells (black) of naturally P(BCMA)_B*18_-presenting MM.1S target cell line at various effector-to-target cell ratios in comparison to MV4–11 P(BCMA)_B*18_-negative target cell line. P(BCMA)_B*18_-unspecific effector T cells (gray) showed no peptide-specific lysis of the same targets. Results are shown as mean ± SEM for three independent technical replicates. Significance was determined using two-tailed paired student’s *t*-test. FSC forward scatter, UPN uniform patient number, n.s. not significant, **p < 0.01, ***p < 0.001.

Next, we aimed to overcome the reported profound immune defects (including impaired function of immune effector cells) in MM^22^, and therefore evaluate the potential of P(BCMA)_B*18_ to induce de novo functional T-cell responses using T cells of MM patients that displayed no preexisting memory immune responses against P(BCMA)_B*18_ following 12-day recall stimulation. While no P(BCMA)_B*18_-specific T cells could be induced in MM-derived samples using our standard priming protocol, we observed de novo induction of P(BCMA)_B*18_-specific T cells with frequencies of 0.2–4.1% (mean 1.6%) within the CD8^+^ T-cell population of an MM patient upon addition of CTLA-4 and PD-1 blocking antibodies (Fig. 4a). These P(BCMA)_B*18_-specific T cells were multifunctional as demonstrated by IFNγ and TNF production as well as upregulation of CD107a (Fig. 4b) and peptide-specific lysis of autologous P(BCMA)_B*18_-loaded CD8^-^ cells (Fig. 4c–e). Moreover, MM-derived P(BCMA)_B*18_-specific CD8^+^ T cells showed specific lysis of MM.1S cells (Fig. 4f–h), which naturally present P(BCMA)_B*18_ as detected by MS-based immunopeptidomics.

Taken together, we identified a naturally presented myeloma-associated, BCMA-derived peptide, which constitutes a promising and highly immunogenic target for tailored T-cell-based immunotherapy and monitoring of immunotherapeutic approaches in MM and other B-cell malignancies.

## Discussion

Valid antigen targets are the main prerequisite for the development of clinically effective antigen-specific cancer immunotherapy. These highly promising target structures are represented by HLA-independent surface antigens, such as BCMA in MM. However, the number of such antigens is limited due to the required exclusive presentation on the surface of tumor cells^23^. HLA-dependent antigens, in contrast, can originate from any intracellular protein or domain and are not restricted to cell surface proteins. Therefore, the amount of potential HLA-dependent targets for a given tumor entity is expected to be considerably higher. In recent years, numerous studies have defined HLA ligands derived from tumor-specific mutations as main targets of immune checkpoint inhibitor-induced T-cell responses in solid tumors^24,25^. Nonetheless, only a very small proportion of DNA-level mutations results in naturally presented, mutation-derived neoantigens detectable in the HLA ligandome^26,27^. Hence, the role of neoantigens for the development of broadly applicable immunotherapy, especially in low mutational burden cancer entities such as MM, remains unclear. We and others have previously described several non-mutated HLA ligands as pathophysiologically relevant targets for T-cell-based immunotherapy approaches^28^, especially for tumors with a low mutational burden, such as hematological malignancies^19,21,29–31^.

In this study we aimed to identify HLA-dependent antigen targets derived from the intracellular domain of the established surface antigen BCMA using an MS-based approach. We identified P(BCMA)_B*18_ as a highly immunogenic naturally presented epitope capable of inducing potent and multifunctional cytotoxic T-cell responses. This is in line with recent data reporting on the high immunogenicity of computationally predicted HLA*02-restricted T-cell epitopes derived from the extracellular surface domain of BCMA^32,33^. Our mass spectrometric approach further allowed us to validate P(BCMA)_B*18_ additionally as a target for CLL, for which plasma BCMA-levels were described as a prognostic factor^34^. However, so far less data are available demonstrating the utility of BCMA-based immunotherapeutic approaches in CLL. As already shown on gene and protein expression level presentation of BCMA-derived peptides is not restricted to myeloma and CLL cells but is—albeit less frequently—detectable in benign B-cell containing tissues^35^. This is in line with other B-lineage-specific targets such as CD19, for which antigen-specific therapies, including CAR T cells and bispecific antibodies, are already approved for clinical use^36,37^. As the expression of CD19 is not limited to malignant B cells, targeting of CD19 can lead to on-target/off-tumor side effects including for example hypogammaglobulinemia, which however are manageable in the clinical setting^38^.

Our approach for the identification of BCMA-derived T-cell epitopes might be translated to other MM-associated antigens such as SLAMF7, CD38, CD74, or CD138^39^, providing naturally processed HLA-presented ligands derived from intracellular domains of established membrane-bound tumor-associated antigens. Such HLA ligands represent promising targets for (i) low side effect single agent vaccine approaches in elderly patients or early disease states^21,28,40^, (ii) TCR-engineered T cells^41^, (iii) antibody-based approaches^42^, or (iv) novel approaches combining HLA-dependent and -independent antigens and treatments^43^. In addition, such T-cell epitopes can be used for the assessment and monitoring of T-cell responses following various types of antigen-specific, or as in this example BCMA-specific, immunotherapy approaches. However, the HLA allotype restriction of HLA-peptide targets, in terms of HLA-B*18 covering only about 12% of the world population^44^, represents a limitation concerning the development of broadly applicable immunotherapy approaches. This calls for combinatorial approaches using peptides of various HLA restrictions or for patient (group)-individualized approaches. However, in this context it has to be taken into account, that different HLA ligands with distinct HLA allotype restrictions derived from the same source protein can show different tissue distribution as demonstrated exemplarily for the P(BCMA)_B*40_ peptide in this study. The lack of correlation between protein expression and the immunopeptidome can be explained by the differential protein processing in malignant cells along with the complexity of HLA ligand formation, which is frequently altered in tumor cells^45,46^.

Besides the selection of optimal target antigens for the development of clinically effective T-cell-based immunotherapies in MM, one must consider the profound immune defects that prevail in these patients and might impair the efficacy of inducing antigen-specific T-cell responses in vivo^22,47^. A major immunosuppressive mechanism in MM is the upregulation of inhibitory immune checkpoint molecules on T cells^22,39^. By employing PD-1/CTLA-4 blockade, we were able to overcome this mechanism and to induce multifunctional cytotoxic P(BCMA)_B*18_-specific T cells, even in MM-derived samples. These data are in line with reports from other solid tumors and hematological malignancies^48–50^, pointing towards a combination of antigen-specific immunotherapy with general approaches optimizing immune responses, as exemplified with immune checkpoint blockade^51,52^.

Taken together, this study provides first evidence that intracellular domain-derived HLA ligands from MM-associated membrane antigens can provide a novel category of highly immunogenic antigen targets for tailored combinatorial immunotherapies for patients suffering from MM or other B-cell malignancies.

## Methods

### Patients, blood samples, and cell lines

PBMCs collected from MM patients at the Department of Hematology and Oncology (Tübingen, Germany), and from healthy volunteers, were isolated by density gradient centrifugation using Biocoll (Biochrom GmbH, Berlin, Germany). Informed consent was obtained from all subjects in accordance with the Declaration of Helsinki protocol. The study was performed according to the guidelines of the local ethics committee. Patient characteristics are provided in Supplemental Table 3. The MM.1S (ATCC CRL-2974) and MV4-11 (DSMZ ACC102) cell lines were tested for mycoplasma contamination and cultured in the recommended cell media as described previously^20^.

### MS data reevaluation

Reprocessing and reanalyzing of previously acquired and published MS data of primary MM patient samples and MCLs^19,20^ was performed as described before^53^. The Proteome Discoverer (v1.3, Thermo Fisher Scientific, Waltham, MA, USA)^54^ was used to integrate the search results of the SequestHT search engine (University of Washington, Seattle, WA, USA) against the human proteome (Swiss-Prot database^55^) without enzymatic restriction. Precursor mass tolerance was set to 5 ppm and fragment mass tolerance to 0.5 Da. Oxidized methionine was allowed as dynamic modification. The false discovery rate, estimated by the Percolator algorithm 2.04^56^, was limited to 5% for HLA class I and 1% for HLA class II. Peptide lengths were set to 8–12 or 8–25 amino acids for HLA class I and II, respectively. HLA class I annotation was performed using SYFPEITHI 1.0^57^ and NetMHCpan4.0^58^.

### Spectrum validation

Spectrum validation of the experimentally eluted peptide was performed by computing the similarity of the spectrum with the corresponding synthetic isotope-labeled peptide measured in a complex matrix. The spectral correlation coefficient was calculated between the spectra of the eluted and the synthetic peptide considering the mass shift of ions containing the isotope-labeled amino acid^59^. Peptides were synthesized as described previously^30^.

### aAPC priming of naïve CD8^+^ T cells

Priming of cytotoxic T lymphocytes was conducted using aAPCs following magnetic-activated cell-sorting (MACS) for CD8^+^ cells as described previously^30^. In detail, 800 000 streptavidin-coated microspheres were loaded with 200 ng biotinylated HLA:peptide monomer and 600 ng biotinylated anti-human CD28 monoclonal antibody (mAb, clone 9.3, in-house production). These aAPCs were used for the stimulation of 1 × 10^6^ CD8^+^ T cells in four stimulation cycles in addition to 5 ng/mL of IL-12 (PromoKine, Heidelberg, Germany). For MM patient-derived CD8^+^ cells the stimulation protocol comprised addition of 1 µg/mL purified anti-human PD-1 (CD279, #14-2799-80) and CTLA-4 (CD152, #16-1529-82, Invitrogen, Carlsbad, CA, USA) mAbs directly after PBMC isolation, after MACS, as well as for the first and third aAPC stimulation.

### Cytokine and tetramer staining

Frequencies of peptide-specific CD8^+^ T cells were determined by tetramer staining using the PE/Cy7 anti-human CD8 mAb (#737661, Beckman Coulter, Brea, CA, USA) and 5 µg/mL of PE-labeled HLA:peptide tetramer (in-house production). Tetramers of the same HLA allotype containing irrelevant control peptides were used as negative control (Supplemental Table 2).

Functionality of peptide-specific T cells was analyzed by ICS using PE/Cy7 anti-human CD8, PacificBlue anti-human TNF (#502920), FITC anti-human CD107a (#328606, BioLegend, San Diego, CA, USA), and PE anti-human IFNγ (#506507, BD, Franklin Lakes, NJ, USA) antibodies as described previously^30^.

Results of tetramer staining were considered positive if the frequency of peptide-specific CD8^+^ T cells was ≥0.1% of viable cells and at least three-fold higher than the frequency of peptide-specific cells in the negative control according to the harmonization guidelines for HLA-peptide multimer assays of the international Cancer Vaccine Consortium proficiency panel^60^. The same evaluation criteria were applied for ICS. All samples were analyzed using a FACS-Canto^TM^ II cytometer and FlowJo software version 10.0.7 (BD).

### Cytotoxicity assays

Peptide-specific CD8^+^ T cells were analyzed for their capacity to induce peptide-specific target cell lysis in the flow cytometry-based VITAL assay^30^. Autologous CD8^-^ target cells were either loaded with the P(BCMA)_B*18_ peptide or an irrelevant control peptide (Supplemental Table 2) and labeled with CFSE or FarRed (life technologies, Carlsbad, CA, USA), respectively. The P(BCMA)_B*18_-specific effector cells were added in the indicated effector-to-target ratios. Specific lysis of peptide-loaded CD8^-^ target cells was calculated relative to control targets. Additionally, we performed the VITAL assay using the P(BCMA)_B*18_-presenting MM.1 S cell line stained with CFSE as target cells. The HLA-B*18^+^ acute myeloid leukemia cell line MV4–11, negative for P(BCMA)_B*18_ in mass spectrometry-based HLA ligandome analysis, was stained with FarRed and served as negative target cell line.

### Amplification of peptide-specific T cells and IFNγ ELISPOT assay

PBMCs of MM patients and HVs were pulsed with 1 μg/mL per peptide and cultured for 12-days. In addition, cells were supplemented on day 3, 5, and 7 with 20 U/mL IL-2 (Novartis, Basel, Switzerland). On day 12, PBMCs were analyzed in the IFNγ ELISPOT assay as described previously^30^ using an ImmunoSpot S5 analyzer (CTL Europe GmbH, Bonn, Germany). The negative control peptide P(negative)_B*18_ (Supplemental Table 2) was used to exclude unspecific immune responses, for example from NK cells. Preexisting memory T-cell responses were considered positive if ≥10 spots/well were counted and the mean spot count was at least three-fold higher than the mean spot count of the negative control.

## Supplementary information

Supplement
