# Supplementary material for: Mass spectrometry-based identification of a B-cell maturation antigen-derived T-cell epitope for antigen-specific immunotherapy of multiple myeloma
Source: Blood Cancer J. 2020 Feb 28;10(2):24. doi: 10.1038/s41408-020-0288-3 (PMC7048774; doi:10.1038/s41408-020-0288-3)
Supplement: Supplementary file 1 — Supplement [file 41408_2020_288_MOESM1_ESM.pdf]

Supplemental Table 1: Results overview of T-cell experiments using cells of healthy volunteers

Healthy volunteer ID	Mean frequency of $P(BCMA)_{B*18}$-specific $CD8^+$ T cells	Multifunctionality of peptide-specific T cells
HV01	0.76%	$IFN\gamma^+$ TNF^+ $CD107a^+$
HV02	0.71%	$IFN\gamma^+$ TNF^+ $CD107a^+$
HV03	0.40%	$IFN\gamma^+$ TNF^+ $CD107a^+$
HV04	0.22%	$IFN\gamma^+$ TNF^+ $CD107a^+$
HV05	2.01%	$IFN\gamma^+$ TNF^+ $CD107a^+$
HV06	0.99%	not tested
HV07	1.42%	not tested
HV08	0.74%	not tested
HV09	0.40%	not tested
HV10	0.42%	not tested

Results of T-cell-based assays using cells of HVs. Mean frequency of *de novo* induced $P(BCMA)_{B*18}$-specific $CD8^+$ T cells using *in vitro* aAPC-based priming experiments and assessment of cytokine production as well as CD107a expression using intracellular cytokine staining of peptide-specific cells. Abbreviations: ID, identification; HV, healthy volunteer.

Supplemental Table 2: Peptides used for T-cell experiments

Peptide ID	Gene name of source protein	Peptide sequence	Position	HLA restriction
P(BCMA)$_{B*18}$	BCMA	DEIILPRGL	111 - 119	B*18
P(negative)$_{B*18}$	ANM1	DEVRTLTY	69 - 76	B*18
P(positive)$_{B*18}$	MUC16	TETEAIHVF	9 507 - 9 515	B*18

HLA-B*18-restricted BCMA-derived peptides as well as positive and negative control peptides used for IFNγ ELISPOT assays and aAPC-based *in vitro* priming experiments. Abbreviations: ID, identification; aAPC, artificial antigen-presenting cell.

Supplemental Table 3: Patient characteristics

UPN	Experiment	Sex	Age	ISS stage	Durie & Salmon	Cytogenetic risk	HLA class I typing
1	E, P	male	72	1	3A	unknown	A*03:01, A*33:01, B*14:02, B*18:01
2	E	male	74	1	2A	low-risk	A*24:02, A*66:02, B*15:09, B*18:01
3	E	female	73	3	3B	high-risk	A*02:01, A*11:01, B*15:01, B*18:01
4	E, P	male	53	3	3A	high-risk	A*03:01, A*11:01, B*18:01, B*35:01

Patients included in T-cell-based assays. Age is given in years at time of sample collection. Cytogenetic risk was assessed according to the International Myeloma Working Group (IMWG) risk stratification (Chng *et al.*, 2014, Leukemia). Abbreviations: UPN, uniform patient number; E, IFNγ ELISPOT; P, aAPC-based priming; ISS, International Staging System.
